# Genomic signatures of cold adaptation in a Himalayan drosophilid

**DOI:** 10.1093/g3journal/jkag190

**Published:** 2026-09-03

**Authors:** Avirup Chakraborty, Subhash Rajpurohit

**Affiliations:** Division of Biological and Life Sciences, School of Arts and Sciences, Ahmedabad University, Commerce Six Road, Navrangpura, Ahmedabad, Gujarat 380009, India; Division of Biological and Life Sciences, School of Arts and Sciences, Ahmedabad University, Commerce Six Road, Navrangpura, Ahmedabad, Gujarat 380009, India

**Keywords:** cold-adapted, Himalayas, *Drosophila nepalensis*, whole-genome sequencing, thermal preference, comparative genomics, genome assembly

## Abstract

*Drosophila nepalensis* is a cold-adapted drosophilid endemic to the Himalayan region. Its ability to survive in harsh, cold conditions makes it a valuable *Drosophila* model for investigating how adaptation to thermal extremes may influence species persistence under future climate change. Here, we report the first de novo genome assembly of *D. nepalensis*, based on a hybrid sequencing strategy that combines Illumina short reads and Oxford Nanopore long reads. Illumina sequencing generated 49.88 million 150 bp paired-end reads (∼14.96 Gbp), while Nanopore sequencing produced 1.35 million long reads totaling ∼0.76 Gbp. The assembled genome spanned ∼178 Mb with an N50 of 83.6 kb and 98% BUSCO completeness, comparable to other well-annotated *Drosophila* genomes. Annotation identified 10,560 protein-coding genes, including transcription factor–rich and stress-related domains such as zinc fingers, WD40 repeats, and ankyrin motifs. Comparative orthology analysis across 6 *Drosophila* species identified 14,168 orthologous clusters, of which 9,173 were shared among all 6 species, indicating a conserved core genomic set across the sampled taxa. *D. nepalensis* showed 83 unique orthogroups and 50 singletons, suggesting some lineage-specific gene expansions associated with cold adaptation and endemicity, including families encoding caspase-family apoptotic regulators, chromatin remodeling proteins (HMGB/protamine-like), and SNARE-domain vesicle trafficking factors. Gene family evolution analysis revealed the highest expansions in the cold-tolerant Himalayan drosophilid, *D. nepalensis*, including significant expansions in serine protease, chaperone, and neurotransmitter transporter families, alongside dramatic contractions of core histone gene families, suggesting lineage-specific chromatin remodeling and ecological specialization.

## Introduction

Montane ecosystems are one of the most vulnerable habitats to climate change, experiencing accelerated rates of temperature rise compared to lowland regions (Pepin et al. 2015). As temperatures rise, species living in high-altitude habitats are increasingly forced to shift their distributions upslope in search of suitable thermal niches (Chen et al. 2011). Such events pose an acute risk of habitat loss and extinction, especially for cold-adapted endemics with narrow elevational ranges and limited dispersal capacities (La Sorte and Jetz 2010; Freeman et al. 2018). Understanding the genetic basis of adaptation in such species is therefore essential for predicting their responses to rapid environmental change.

*Drosophila nepalensis* is a less-known drosophilid endemic to the Western Himalayan region. Its distribution was earlier confined to the mid-altitudes, but has now been shown to move upslope in search of suitable habitats (Parkash et al. 2013). The Himalayas have undergone substantial anthropogenic load in recent decades, with mean temperatures increasing at nearly twice the global average (Shrestha et al. 2012). This climatic shift has not just affected *D. nepalensis*, but many other montane insects (Parkash et al. 2005; Rajpurohit et al. 2008; Shah et al. 2012; Singh et al. 2024) in the Himalayas. This increase in temperature raises concern that cold-adapted *Drosophila*, such as the *D. nepalensis* found in the Himalayas, are particularly vulnerable to climate change. Notwithstanding the ecological importance of this species, no genomic resource is available to understand the climate change-driven dynamics of its adaptation across spatial and temporal scales.

Genomic data provide powerful tools for understanding the evolutionary history, demographic responses, and adaptive potential of a species under climatic stresses (Allendorf et al. 2010). In particular, high-quality reference genomes enable the identification of genes underlying tolerance to various abiotic stresses, such as temperature. Thus, the availability of high-quality reference genomes is essential to facilitate population genomic studies of local adaptation and supports predictive modeling of future range shifts (Hoffmann and Sgrò 2011). For drosophilids, such resources have been invaluable in elucidating the molecular mechanisms of thermal adaptation (Adrion et al. 2014). However, these insights have been largely restricted to cosmopolitan species such as *Drosophila melanogaster*, leaving montane specialists, such as *D. nepalensis*, largely uncharacterized. Moreover, the literature is biased toward studies of temperate species and model organisms, and *D. nepalensis* fills that gap.

Here, we present the first de novo genome assembly and gene prediction of *D. nepalensis*, generated using a hybrid approach that combines Illumina short reads and Oxford Nanopore long reads. This high-quality genome serves as a foundational resource for studying the genetic basis of cold adaptation and endemicity in Himalayan drosophilids. We further performed a thermal preference assay to understand the thermal niche of the species, thereby allowing us to select 6 species for a comparative genomics study, ensuring a balance of cold- and warm-adapted species and close relatives. This will enable future population genomics, functional genomics, and comparative analyses studies in addition to supporting efforts to understand and predict the evolutionary trajectories of cold-adapted species in response to rapid climate change.

## Materials and methods

### Collection of flies

*D. nepalensis* females were collected using bait traps from Jhalla, Uttarakhand (31.0278223, 78.7150254, 2,439 m asl). Each collected female was placed in an individual vial with standard food to allow oviposition. These vials were transported to the laboratory and maintained at 23 °C in a 12:12-h light-dark cycle. Vials were checked every day for the presence of eggs or larvae. The vials with no eggs or larvae were discarded, and those with eggs or larvae were allowed to grow until all the flies had eclosed. The males in each viable vial were verified as *D. nepalensis* using standard taxonomic keys (Fig. 1). Among the successful lines, 13 isofemale lines of *D. nepalensis* were selected. Twenty adult males were collected from each line and pooled for genomic DNA extraction.

**Fig. 1.**
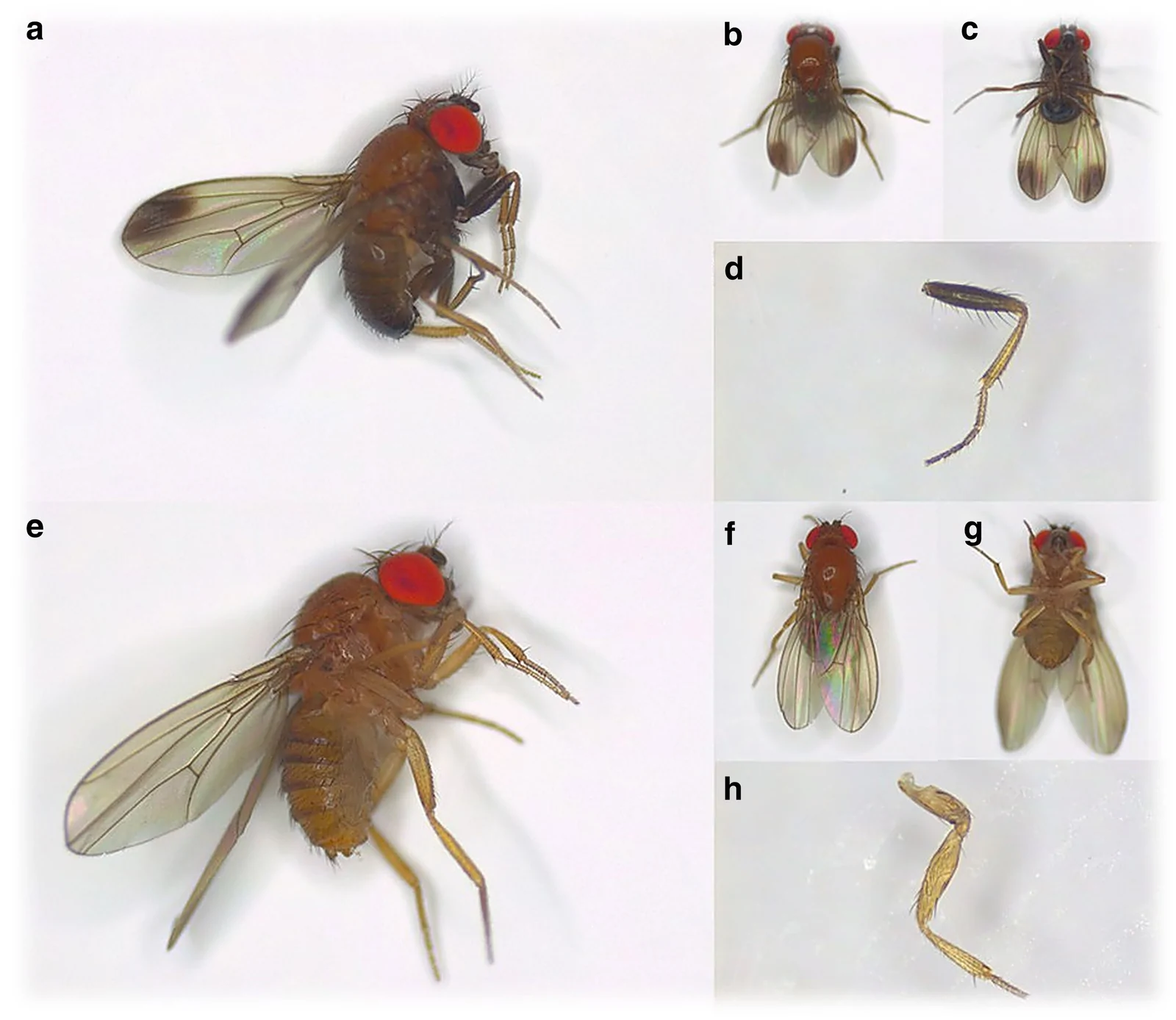
*D. nepalensis*, collected from the wild in the Western Himalayas, on an image plate. a–d) Male, e–h) female, a and e) lateral side, b and f) dorsal side, c and g) ventral side, and d and h) foreleg. The males and females are easily distinguishable, with the male being distinctly pigmented on its body, wing, and foreleg.

### Genomic DNA extraction and quality assessment

Genomic DNA was extracted from adult *D. nepalensis* males using a standard phenol–chloroform method (Hegde et al. 2022). DNA quality was assessed by agarose gel electrophoresis (Green and Sambrook 2019), and quantity was measured using a Qubit 4 Fluorometer (Thermo Fisher Scientific, United States) to ensure suitability for whole-genome sequencing.

### Library preparation and sequencing

For short-read sequencing, Illumina libraries were prepared using standard paired-end library preparation kits. Sequencing was performed on the Illumina platform using 150 bp paired-end (150 × 2) chemistry, generating approximately 90–100× coverage of the estimated genome size. For long-read sequencing, libraries were prepared using the Oxford Nanopore Technologies (ONT) ligation sequencing kit (SQK-LSK114.96; ONT, Oxford, UK) following the manufacturer's protocol. The prepared library was sequenced on the Oxford Nanopore PromethION platform (PromethION P24 and Data Acquisition Unit) using FLO-PRO114M flow cells, generating approximately 25–30× coverage. Raw sequencing data from both platforms were provided via FTP.

### Basecalling and quality control

Nanopore raw FAST5 data were basecalled using Guppy (ONT) in high-accuracy mode to generate FASTQ files. Low-quality Nanopore reads (average Q < 7 or < 1 kb in length) were filtered using Filtlong, and quality statistics were examined with NanoPlot. Adapter trimming was performed with Porechop. Illumina reads were quality-checked using FastQC and trimmed for adapters and low-quality bases using CutAdapt.

### Hybrid genome assembly and polishing

Filtered Nanopore long reads were assembled de novo using Flye (Freire et al. 2022) with default parameters. The draft assembly was polished in 2 rounds with Racon (Vaser et al. 2017) and one round of Medaka by Oxford Nanopore Technologies using Nanopore reads. Subsequently, Illumina reads were aligned to the polished assembly, and further polishing was performed using 2 rounds of Pilon (Chen et al. 2021) to correct residual basecalling errors and small indels, thereby producing a high-accuracy consensus assembly.

### Assembly evaluation

Assembly quality was assessed using QUAST (Gurevich et al. 2013) to obtain standard assembly metrics, including genome size, contig N50, and GC content. Genome completeness was evaluated with BUSCO (Simão et al. 2015) using the insecta_odb10 lineage dataset. Read mapping rates for both Illumina and Nanopore datasets were calculated to verify completeness and structural accuracy.

### Contaminant screening

To minimize the inclusion of exogenous DNA, the assembly was screened for contamination using FCS-GX (Astashyn et al. 2024). Classification was performed against the standard prebuilt FCS-GX database, and all reads assigned to prokaryotes and other non-*Drosophila* eukaryotes were removed. This procedure ensured that the assembly and downstream repeat library construction were based on a dataset filtered for putative contaminants.

### Genome annotation

Repetitive elements were identified as part of the MOSGA2 workflow (Martin et al. 2021). A de novo repeat library was constructed using RepeatModeler (Smith et al. 2007), and repetitive sequences were subsequently annotated and soft-masked using RepeatMasker (Smith et al. 2007). This masked assembly was used as input for downstream gene prediction to prevent spurious gene model inference from repetitive regions. Structural gene annotation was carried out using MOSGA2, which integrates multiple ab initio and evidence-based predictors. Gene models were predicted using AUGUSTUS and guided by protein homology evidence from the model organism, *D. melanogaster*. MOSGA2 merged and filtered redundant or low-confidence predictions to produce a high-confidence, non-redundant gene set.

### Functional annotation

Predicted protein-coding genes were functionally annotated using MOSGA2's functional annotation module. Protein domains and Gene Ontology (GO) terms were assigned using InterProScan. Non-coding RNAs were identified using tRNAscan-SE (for tRNAs) and with the Rfam database (for rRNAs and snRNAs). A subsequent InterPro scan was performed to identify the most enriched protein domains and GO terms. All annotation outputs (GFF3, protein FASTA, CDS FASTA, and functional annotations) were generated and compiled using MOSGA2 and InterPro. Quantification and visualization of all downstream analyses were done using ggplot2 (Wickham 2016).

### Thermal preference assay

A thermal preference assay was performed for 3 species, *D. nepalensis* (cold-adapted) (Parkash et al. 2005), *D. melanogaster* (cosmopolitan) (Markow 2015), and *Drosophila ananassae* (cosmopolitan and warm-adapted) (Singh 2025) to lay the groundwork for genomic comparisons of different thermal adaptations. The thermal preference assay was performed on a custom-built thermal gradient apparatus in the laboratory according to the method described here (Chakraborty and Rajpurohit 2026). A thermal gradient was set up on an aluminum plate (dimensions, length: 45 cm × breadth: 16 cm × height: 1 cm). A temperature gradient was created by circulating cold and hot water at the extreme ends of the aluminum plate. An insulated Plexiglas compartment was created within the aluminum plate to house 10 glass tubes (30 cm in length, 0.3 cm in inner diameter). The gradient was allowed to stabilize at 14 °C at the cold end and 32 °C at the hot end. The entire setup was housed at a laboratory temperature of 25 °C. The gradient was standardized using temperature probes at different intervals along its length. A black fabric cabinet covered this entire apparatus to prevent light from entering the gradient setup. All the experiments were run in the dark. A total of 10 individuals of either sex were collected from each of the 10 isofemale lines of each species (*D. nepalensis*, *D. melanogaster*, and *D. ananassae*) and kept in a fresh food vial for 24 h before the assay. These individuals were then introduced into a thin cylindrical glass tube using a funnel and plugged at both ends with cotton. These tubes were prepared and placed on the gradient tube loading cabinet. The flies were then allowed to move to their preferred zones, and a photograph of the gradient was captured from the top with a webcam installed inside the dark cabinet. The image was then analyzed by dividing the gradient into cold, medium, and hot zones. The number of flies in each zone for each tube was calculated. The samples were run between 10:00 AM and 8:00 PM on a single day.

### Statistical analysis

The thermal zone preferences across these 3 *Drosophila* species (*D. nepalensis*, *D. melanogaster*, and *D. ananassae*) were analyzed using non-parametric statistical tests due to the non-normal distribution of individual counts per line. We first conducted Kruskal–Wallis H-tests to assess overall differences in individual distribution across thermal zones (cold: 14–20 °C, medium: 20–26 °C, hot: 26–32 °C) within each species. Subsequently, post hoc pairwise comparisons between zones were performed within each species using Wilcoxon rank-sum tests, with *P*-values adjusted for multiple comparisons using the Benjamini–Hochberg method. Statistical significance was set at α = 0.05. All analyses were conducted in R (version 4.5) using the rstatix (Kassambara 2019) and dplyr (Yarberry 2021) packages.

### Comparative genomics and orthologous cluster identification

To investigate the evolutionary relationships and shared gene content between *D. nepalensis* and related species, a comparative genomics analysis was conducted using OrthoVenn3 (Sun et al. 2023), employing the OrthoFinder algorithm for ortholog clustering. Six *Drosophila* species were included in the analysis: *D. nepalensis*, which has a de novo assembled genome and is endemic to the Himalayan region and adapted to cold environments; *Drosophila rufa*, another cold-adapted species, serving as a closely related reference for high-altitude adaptation (Xue et al. 2019); *D. melanogaster*, a cosmopolitan model species (Markow 2015), included as a representative of temperate-to-tropical environments and as a well-annotated genomic reference; *D. ananassae*, cosmopolitan and warm-adapted (Yadav and Singh 2005), providing contrast against high-altitude cold specialists; *Drosophila takahashii*, a species from tropical and subtropical habitats (Kimura et al. 1994), distributed across East and South Asia; and *Drosophila suzukii*, a cold-tolerant species with an invasive range expansion, into temperate climates (Jakobs et al. 2015).

The inclusion of these species enabled a comparative framework encompassing cold-adapted Himalayan species, other cold-adapted species, and cosmopolitan species, allowing the assessment of shared and unique orthologous gene clusters potentially associated with cold adaptation and endemism. All 6 species belong to the family Drosophilidae and the genus *Drosophila*, providing a phylogenetically coherent framework for comparative genomic analysis.

For all 6 species, protein-coding gene prediction was performed using AUGUSTUS (v3.5) with the *D. melanogaster* gene model, generating predicted protein FASTA files from the assembled genomes (de novo assembly of *D. nepalensis* and assembled genomes from NCBI). This was done to maintain uniformity in comparisons across the 6 species, even though proteomes were available for only 4 (*D. melanogaster*, *D. ananassae*, *D. suzukii*, and *D. takahashii*) in NCBI. All protein FASTA sequences were uploaded to OrthoVenn3 and analyzed using default parameters with the OrthoFinder clustering algorithm. Orthologous cluster numbers, shared and unique gene families, GO enrichment analyses, and phylogenetic relationships were computed within OrthoVenn3. Average nucleotide identity (ANI) and BUSCO completeness for all the genome assemblies of the 6 species were also conducted using MOSGA2 (Martin et al. 2021). Subsequent visualizations were generated.

### Functional characterization of gene family sets

Functional annotation of gene families was performed using InterProScan results generated for *D. nepalensis* protein sequences (Supplementary File 4). Gene family membership for each CAFE-significant orthogroup was extracted by mapping cluster identifiers from the CAFE output to the orthogroup membership file (Orthogroups.txt) produced by OrthoVenn3. Four gene sets were characterized: (i) gene families exclusive to *D. nepalensis* (present in *D. nepalensis* but absent in all 5 other species); (ii) gene families significantly expanded in *D. nepalensis* (CAFE5 *P* < 0.05, positive change); (iii) gene families significantly contracted in *D. nepalensis* (CAFE5 *P* < 0.05, negative change); and (iv) gene families with shared expansion or contraction events among cold-adapted species (*D. nepalensis*, *D. suzukii*, and *D. rufa*), classified as cold-adapted based on their high-altitude or temperate distribution and documented cold tolerance. For each gene set, the InterPro domain composition of *D. nepalensis* member genes was tabulated, and the most frequently occurring domains were reported.

The entire pipeline, from DNA extraction to comparative genomics, has been summarized in Fig. 2.

**Fig. 2.**
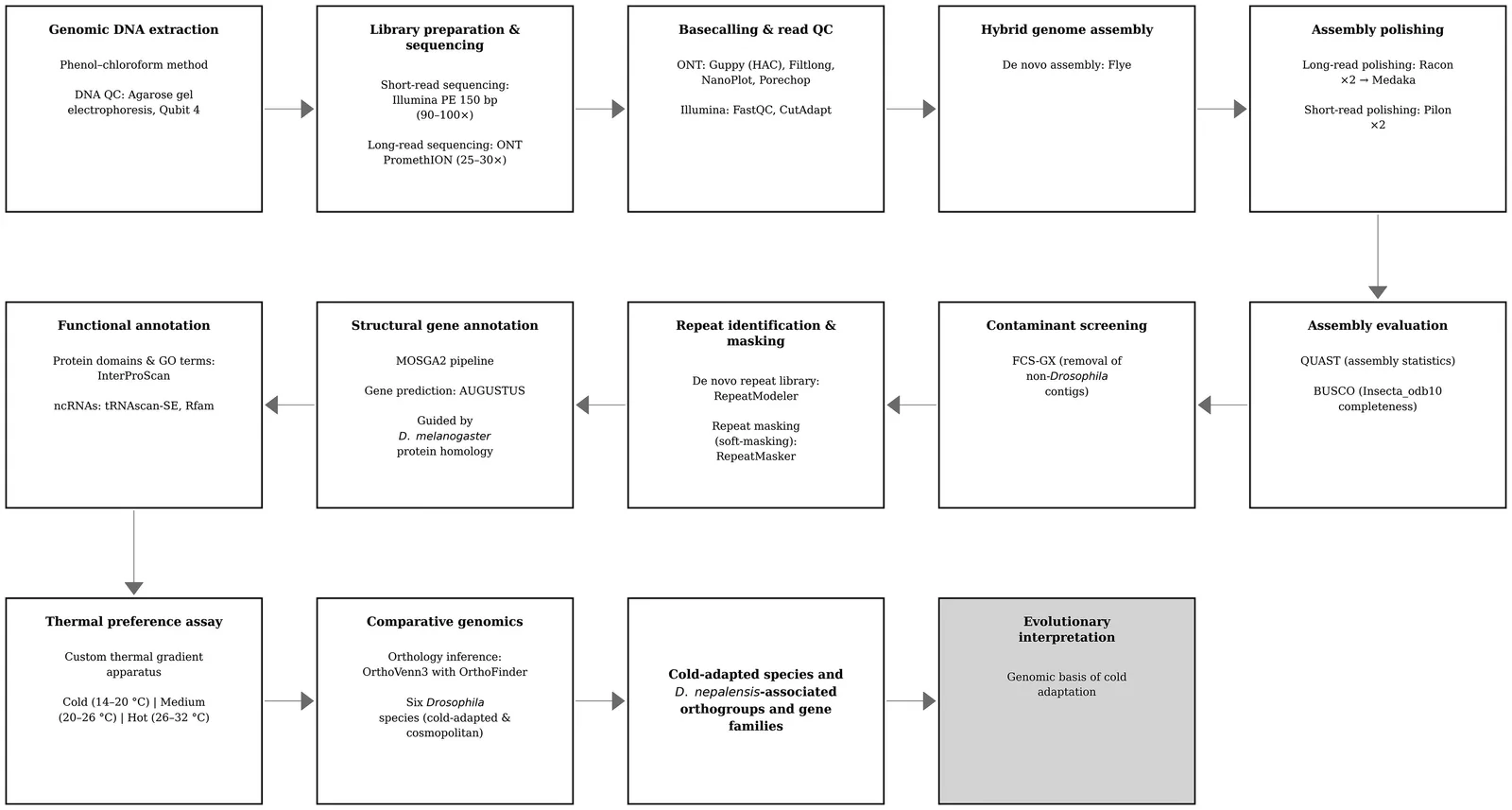
Pipeline for whole-genome sequencing, analysis, and annotation followed by phenotyping and comparative genomics.

## Results

### Whole-genome sequencing and assembly

Whole-genome sequencing of *D. nepalensis* generated both short-read Illumina and long-read Oxford Nanopore datasets. Illumina sequencing generated approximately 49.88 million 150-bp paired-end reads, corresponding to roughly 14.96 Gbp of data. Oxford Nanopore sequencing yielded 1,352,750 reads, totaling 757 Mbp. Assuming a genome size of approximately 180–200 Mb (typical for *Drosophila* species), this represents 90–100× Illumina coverage and 25–30× Nanopore coverage, sufficient for generating a high-quality hybrid de novo assembly.

Filtered Nanopore long reads were assembled de novo using Flye, and the resulting draft was iteratively polished with 2 rounds of Racon and one round of Medaka. Subsequently, Illumina short reads were aligned to the polished assembly and corrected using 2 rounds of Pilon to improve base-level accuracy. The final hybrid assembly comprised 5,285 contigs, totaling 178.07 Mb and with a contig N50 of 83.6 kb (Table 1; Fig. 3). The largest contig measured 541 kb and a total GC content of 40.04%. These metrics indicate a contiguous and well-balanced assembly comparable in size and composition to other *Drosophila* genomes.

**Fig. 3.**
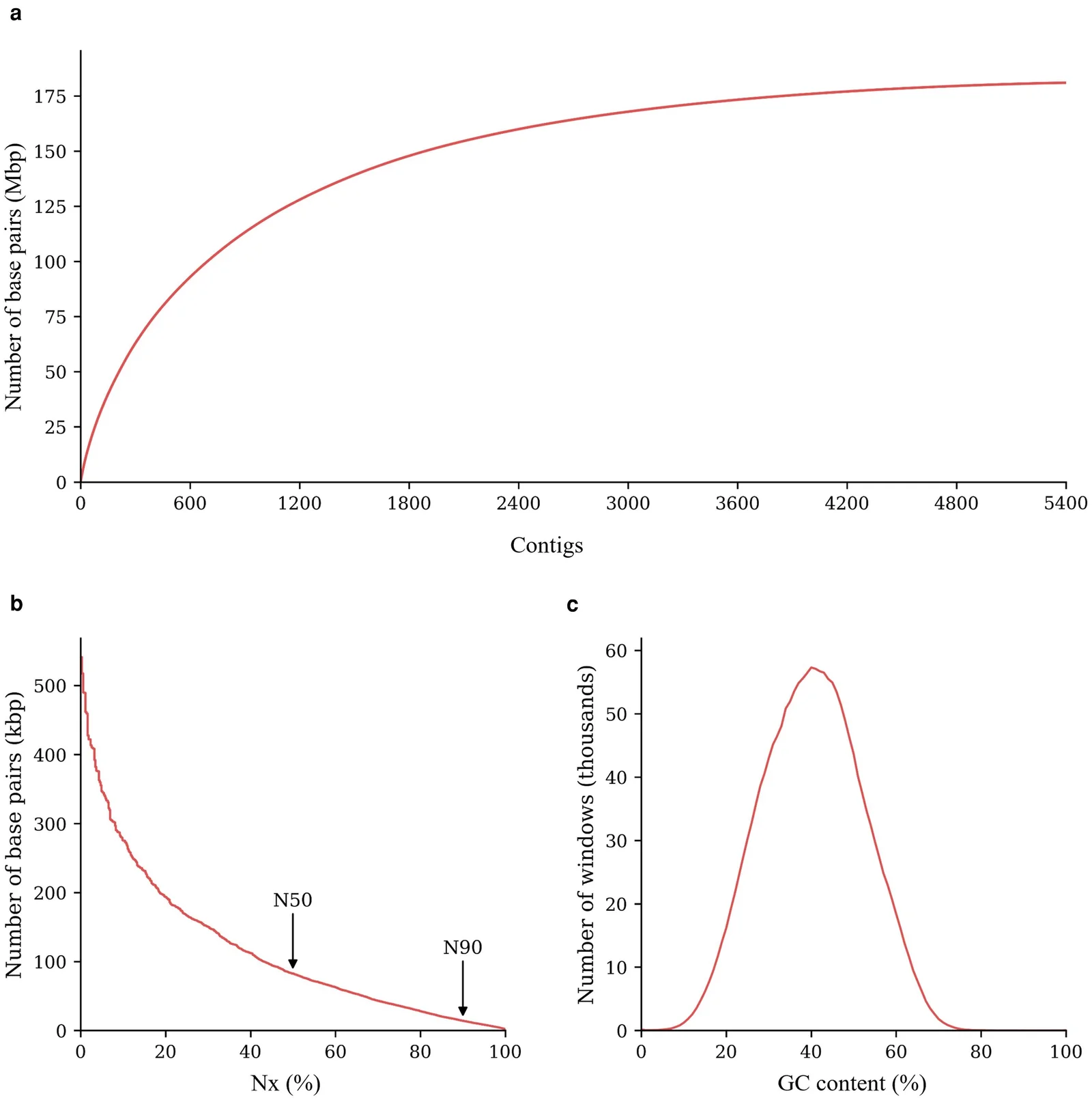
Comprehensive quality assessment of the de novo genome assembly of *D. nepalensis* using QUAST. The plots summarize the assembly’s structural and compositional features. The cumulative length plot (top) shows a gradual increase in total assembly size, with contigs ordered from largest to smallest. The Nx plot (bottom left) shows the N50 and N90 values. The GC content distribution plot (bottom right) shows a unimodal distribution centered at 40.04% GC, consistent with the typical nucleotide composition of *Drosophila* species. No ambiguous bases were detected (0 Ns per 100 kbp), indicating a high-quality, gap-free assembly.

**Table 1.** Summary of genome assembly, repeat annotation, and completeness statistics for *D. nepalensis.*

Characteristic	Value
Assembly statistics (QUAST)	
Total assembly length (bp)	178,067,383
Number of contigs	5,285
Longest contig (bp)	541,192
Shortest contig (bp)	407
Contig N50 length (bp)	83,651
Contig N90 length (bp)	14,121
Number of contigs ≥ N50	554
Number of contigs ≥ N90	2,514
GC content (%)	40.04
Annotation statistics (MOSGA2)	
Number of annotated features	77,140
Number of protein-coding genes	10,560
Average transcript length (bp)	6,168
Average CDS length (bp)	399
Number of tRNA genes	233
Number of rRNA genes	43
Total repeat elements	66,304
BUSCO completeness (insecta_odb10)	
Complete BUSCOs (C)	98%
Single-copy (S)	94.3%
Duplicated (D)	3.7%
Fragmented (F)	0.9%
Missing (M)	1.2%
BUSCOs with internal stop codons	48

The final assembly spans ∼178 Mb across 5285 contigs, with an N50 of 83.6 kb and a GC content of 40.04%. A total of 10,560 protein-coding genes, 233 tRNAs, and 43 rRNAs were annotated, alongside 66,304 repeat elements. BUSCO analysis using the insecta_odb10 dataset indicated 98% completeness, reflecting a highly complete genome assembly.

Assembly completeness was assessed using BUSCO v5.8.0 with the insecta_odb10 lineage dataset (*n* = 1,339). The assembly exhibited 98% complete BUSCOs (C), of which 94.3% were single-copy (S) and 3.7% were duplicated (D); 0.9% were fragmented (F), and 1.2% were missing (M) (Fig. 4). These results indicate that the genome assembly captures nearly the entire expected gene set for insects. The absence of gaps and zero ambiguous bases (N's) further reflect high assembly accuracy and completeness.

**Fig. 4.**
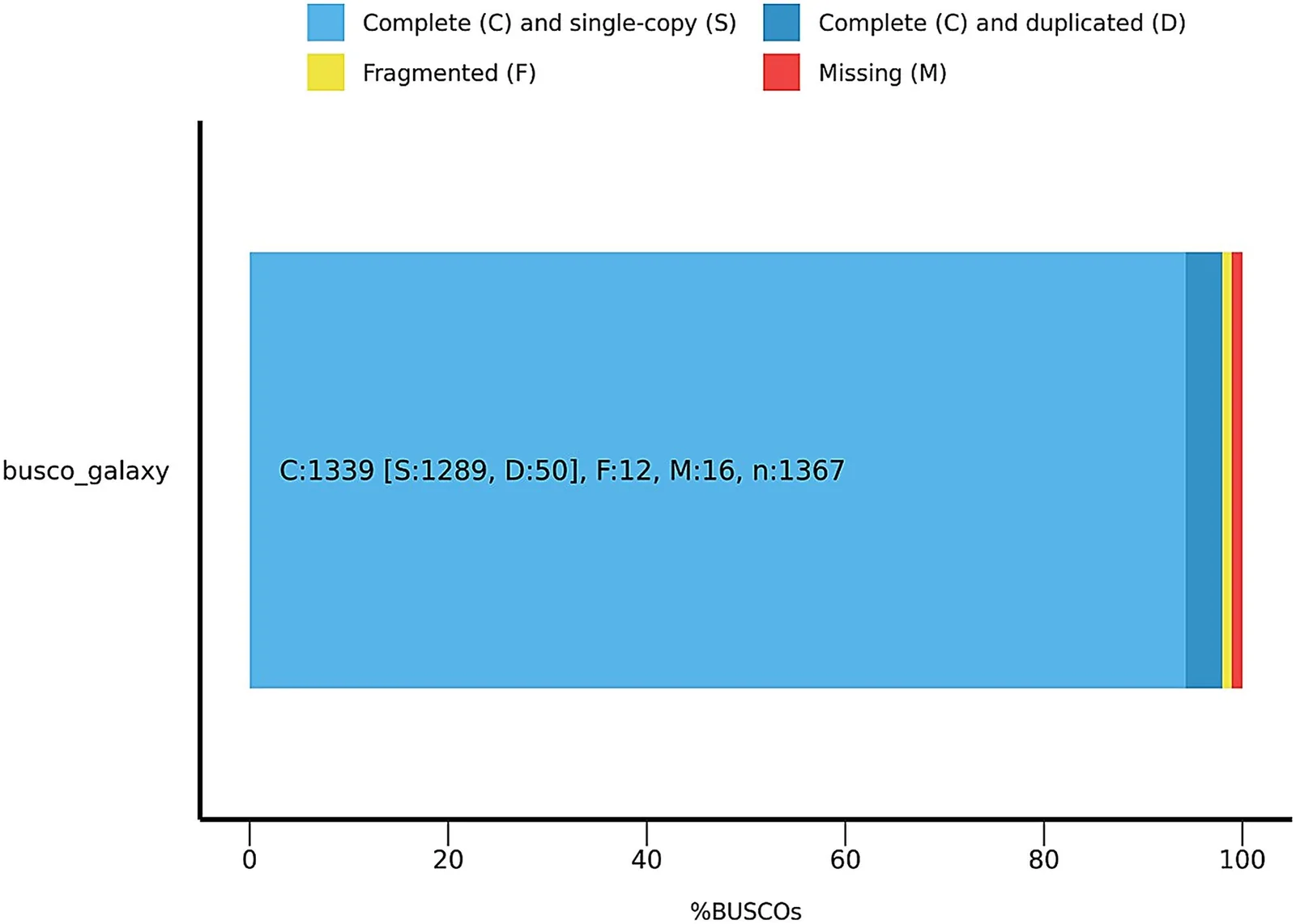
BUSCO assessment of the *D. nepalensis* genome assembly. BUSCO completeness was evaluated with the insecta_odb10 dataset (*n* = 1,339). The assembly contained 98% complete BUSCOs, comprising 94.3% single-copy and 3.7% duplicated genes. Additionally, 0.9% were fragmented, and 1.2% were missing. These results indicate that the assembly captures nearly the entire expected insect gene set, supporting its high completeness and suitability for downstream analyses.

### Contaminant screening, repeat content, and genome annotation

Before annotation, the FCS-GX tool was used to identify and filter out the contaminants. It assigned the vast majority of contigs to *Drosophila*. In contrast, a small subset (less than 5%) was classified as bacterial, predominantly within the family Acetobacteraceae (genera such as *Acetobacter* and *Gluconobacter*), together with low-abundance hits to other bacterial lineages. All contigs classified as non-*Drosophila* were removed prior to repeat discovery with RepeatModeler and downstream annotation, so the repeat landscape and gene content reported here reflect a contaminant-filtered *D. nepalensis* genome.

RepeatMasker analysis of the *D. nepalensis* genome identified that 27.01% of the assembly comprises repetitive elements. Long Interspersed Nuclear Element (LINE) and Long Terminal Repeat (LTR) retrotransposons are predominant, with notable contraction or absence of several DNA transposon families (e.g. PiggyBac, En-Spm, MULE-MuDR), potentially reflecting lineage-specific genome adaptation and contraction of transposable elements (TEs) associated with adaptation to harsh environments. The considerable fraction of unclassified repeats (12.44%) underscores the unique TE landscape in Himalayan drosophilids and points to further evolutionary novelty yet to be characterized. In total, 77,140 genomic features (genes, repeats, and structural RNAs) were annotated. Functional annotation using InterProScan revealed conserved protein domains and high similarity to known *Drosophila* proteins in the Swiss-Prot and NCBI nr databases.

### Structural and functional annotation

Structural annotation of the *D. nepalensis* genome using MOSGA2 identified 10,560 protein-coding genes, with an average transcript length of 6,168 base pairs (bp) and an average coding sequence (CDS) length of 399 bp. Additionally, 233 tRNA genes and 43 rRNA genes were annotated, along with 77,140 annotated genomic features, including repeats and structural RNAs (Table 1).

Functional annotation of the predicted protein sequences using InterProScan yielded 100,862 domain annotations, corresponding to 12,253 unique proteins. These annotations were assigned across 17 distinct databases, including major conserved domain and family repositories such as Pfam, Gene3D, SUPERFAMILY, SMART, and PANTHER, which collectively represented more than 70% of the hits. The distribution of database representation indicated that Pfam contributed the highest number of annotations (20,638 entries), followed by SMART (15,813), and SUPERFAMILY (13,146), reflecting a comprehensive coverage of both structured and disordered protein regions (Supplementary File 1).

Analysis of the most frequently represented InterPro domains revealed that the zinc finger C2H2-type domain was by far the most abundant, occurring in 4,939 proteins, suggesting a substantial repertoire of transcription factors and DNA-binding proteins within the genome. Other highly represented domains included the P-loop containing nucleoside triphosphate hydrolase (1,416), WD40 repeat (1,823), and EGF-like domain (1,375), all of which are commonly involved in regulatory, signaling, and catalytic processes (Supplementary File 1). Additional domains, such as serine protease trypsin domain, ankyrin repeats, immunoglobulin-like folds, fibronectin type III domains, and leucine-rich repeats, were also prevalent, indicating the diversification of proteins involved in cell–cell interactions, signaling, and proteolytic activity.

GO term analysis identified a total of 2,286 unique GO terms (1,044 molecular function, 915 biological process, 327 cellular component) across the annotated proteome. The most frequent GO terms were GO:0005515 (protein binding), GO:0016020 (membrane), GO:0006508 (proteolysis), and GO:0005524 (ATP binding), followed by GO:0004252 (serine-type endopeptidase activity) and GO:0005509 (calcium ion binding). The predominance of these functional categories highlights the importance of binding, catalytic, and membrane-associated activities in the molecular landscape of the species. Together, these findings demonstrate that the proteome is enriched in regulatory, enzymatic, and structural domains, reflecting a functionally complex and evolutionarily versatile genome.

### Comparative genomics

#### Thermal preference comparisons across 3 species

All 3 *Drosophila* species showed significant differences in their distribution across thermal zones (Kruskal–Wallis tests: *D. ananassae*, H = 16.93, *P* < 0.001; *D. melanogaster*, H = 8.32, *P* = 0.016; *D. nepalensis*, H = 26.68, *P* < 0.001; Fig. 5; Supplementary File 2). Post hoc pairwise comparisons revealed a consistent pattern of cold-zone preference across species. *D. ananassae* showed significantly higher occupancy in cold zones (median = 5.0 individuals per line) compared to both medium (median = 2.5, *P* = 0.004) and hot zones (median = 2.0, *P* < 0.001), with no significant difference between medium and hot zones (*P* = 0.243). *D. melanogaster* exhibited similar trends, with cold zones (median = 5.0) hosting significantly more individuals than hot zones (median = 2.0, *P* = 0.015), though the difference between cold and medium zones was not significant (*P* = 0.343). *D. nepalensis* showed the most pronounced thermal preference, with substantially higher occupancy in cold zones (median = 8.0) than in medium (median = 2.0, *P* < 0.001) or hot zones (median = 1.0, *P* < 0.001), consistent with its high-altitude adaptation. This indicates a cold preference for all species, with the highest cold preference exhibited by *D. nepalensis*. However, the higher distribution of individuals in medium and hot zones in *D. melanogaster* and *D. ananassae*, compared to *D. nepalensis*, suggests a cosmopolitan distribution. This lays the groundwork for understanding the genome of *D. nepalensis* species in relation to these 2 species and 3 other species with different thermal adaptations: *D. rufa* (cold-adapted), *D. takahashii* (warm-adapted), and *D. suzukii* (cold-adapted), for comparative genomics.

**Fig. 5.**
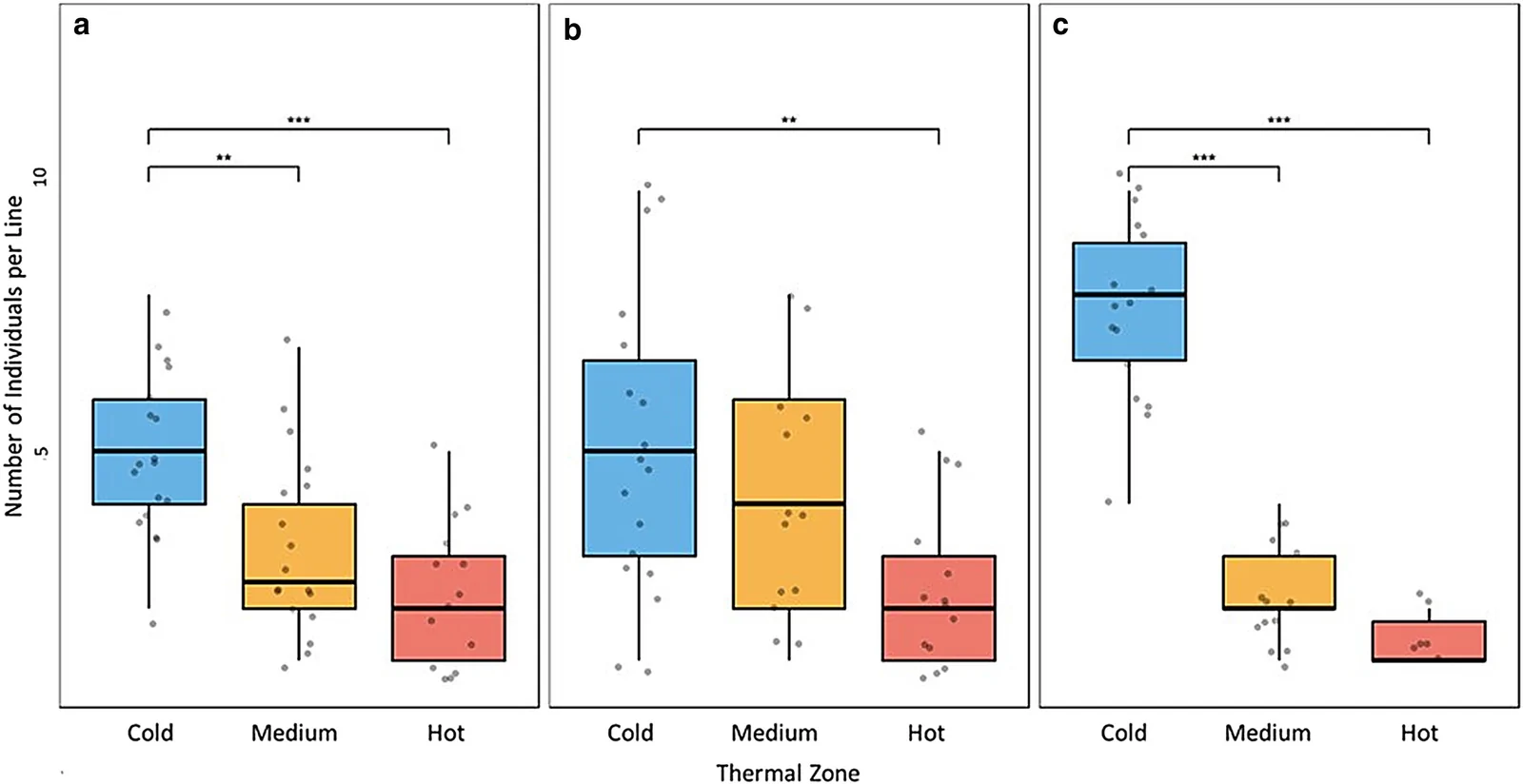
Thermal preference distribution across 3 *Drosophila* species. Box plots showing the distribution of individual counts per line within 3 thermal zones (cold: 14–20 °C, medium: 20–26 °C, hot: 26–32 °C) for *D. ananassae* a), *D. melanogaster* b), and *D. nepalensis* c). Boxes represent the interquartile range, with the median shown as a black line. Individual data points are overlaid as jittered dots. Significant pairwise differences (Wilcoxon rank-sum test, Benjamini–Hochberg corrected) are marked above the boxes: *P < 0.05, **P < 0.01, ***P < 0.001. All 3 species show a significantly higher preference for cold zones than for hot zones, with *D. nepalensis* exhibiting the most pronounced cold preference.

#### Comparative orthology and core genome composition

Comparative orthology analysis using OrthoVenn3 identified 9,173 orthologous clusters shared among all 6 *Drosophila* species (*D. nepalensis*, *D. melanogaster*, *D. ananassae*, *D. suzukii*, *D. takahashii*, and *D. rufa*), representing the conserved core genome within the genus (Fig. 6). This extensive shared gene set highlights the strong evolutionary conservation of developmental, metabolic, and regulatory pathways across *Drosophila*.

**Fig. 6.**
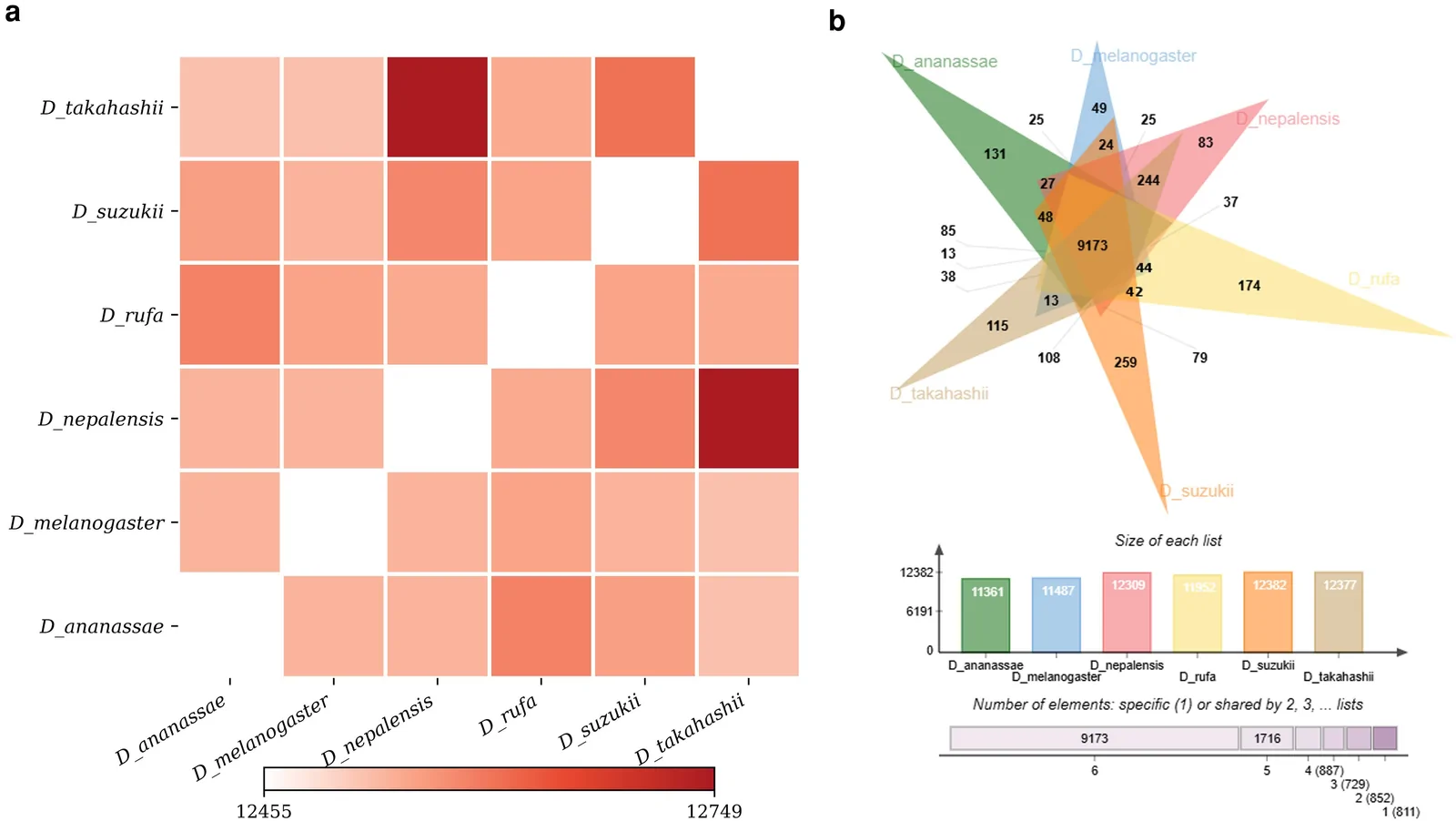
Comparative genomics of 6 Drosophila species, *D. nepalensis*, *D. rufa*, *D. takahashii*, *D. suzukii*, *D. ananassae*, and *D. melanogaster*, and based on their respective protein FASTA sequences. a) Heatmap illustrating orthogroup copy number distribution among 6 *Drosophila* species. Color intensity represents normalized orthogroup copy numbers, with darker shades indicating higher abundance. b) The distribution of orthologous gene clusters identified using OrthoVenn3. The central overlapping region represents clusters shared by all 6 species and constitutes the conserved core genome. Each colored sector denotes species-specific clusters, with the number of unique orthogroups indicated along each ray. The bar plots below summarize the total orthologous cluster counts per species, while the purple horizontal bar at the bottom categorizes the overall distribution of orthogroups—including core (shared by all species), partially shared, and lineage-specific clusters.

In addition to these core orthogroups, 1,716 clusters were shared among subsets of species, indicating lineage-specific retention or loss of gene families during divergence. Notably, *D. nepalensis* and *D. rufa*—both cold-adapted Himalayan taxa—shared a substantial number of orthogroups exclusively with *D. suzukii*, another cold-tolerant lineage. This pattern suggests possible shared ancestry or convergent retention of genes involved in cold adaptation.

Species-specific orthologous clusters ranged from 49 in *D. melanogaster* to 174 in *D. rufa*, with *D. nepalensis* showing 83 unique clusters—fewer than most species in the comparison, suggesting that lineage-specific gene innovation in this species is modest, and that its ecological distinctiveness is more likely reflected in gene family copy number dynamics (assessed by CAFE) than in the gain of wholly novel gene families. Overall, the majority of gene families were shared across taxa, reflecting broad evolutionary stability within the *Drosophila* genus, with limited but functionally significant divergence at the species level—exemplified by the contraction of core histone gene families and the expansion of chromatin-remodeling and stress-response gene families in *D. nepalensis* (see Functional signatures of gene family expansion below).

#### Orthology clustering and gene family composition

Comprehensive analysis of the 6 *Drosophila* proteomes yielded 14,168 orthologous clusters encompassing 94,460 protein sequences (Table 2; Supplementary File 3). Among these, 7,619 single-copy orthologs were identified across all species, forming a robust phylogenomic core suitable for evolutionary inference.

**Table 2.** Summary of orthology clustering statistics across all 6 *Drosophila* species.

Category	Count
Overlaps	63
All clusters	14,168
Single-copy clusters	7,619
All proteins	94,460
All singletons	2,468
Percentage of singletons	2.61%

“All clusters” refers to the total number of orthogroups identified across all species combined. “Single-copy clusters” refers to the subset of orthogroups in which each of the 6 species is represented by exactly one gene copy. “Singletons” are individual protein sequences not assignable to any orthogroup and should not be conflated with species-specific clusters. Singleton counts per species are reported in the Results text.

A total of 2,468 proteins (2.61%) were classified as singletons, representing species-specific or highly divergent genes. These singletons may correspond to recently evolved, rapidly evolving, or annotation-unique loci. Additionally, 63 clusters showed overlaps between orthogroups, suggesting possible gene duplication events or, alternatively, annotation inconsistencies across species.

#### Species-specific clustering patterns

Species-level orthology statistics revealed distinct patterns in gene content and clustering (Table 2). *D. suzukii* encoded the highest number of predicted proteins (18,756), the highest number of clusters (12,382), and the highest number of singletons (57). *D. nepalensis* and *D. rufa* showed comparable singleton counts (50 and 46, respectively), indicative of lineage-specific gene diversification. *D. takahashii* and *D. melanogaster* showed the least singleton counts (20 and 19, respectively), while *D. ananassae* had 52 singletons. These differences suggest subtle but functionally consequential variation in gene family complexity and genome plasticity among the 6 species, as illustrated by the expansion of neurotransmitter transport and mitochondrial oxidative phosphorylation gene families specifically in *D. nepalensis*, and the shared expansion of stress-response chaperones like heat shock proteins (HSPs) between *D. nepalensis* and *D. rufa*.

#### Gene family expansion and contraction dynamics

To further explore evolutionary changes in gene family size, a CAFE-based analysis was performed to estimate gene family expansion and contraction events across the *Drosophila* phylogeny (Fig. 7). The analysis revealed asymmetric patterns of gain and loss among the examined lineages. Ancestral nodes exhibited predominantly contraction-biased dynamics (e.g. node 7: 20 expansions, 169 contractions; node 3: 124 expansions, 214 contractions), indicating that gene family loss has been the dominant evolutionary force across the shared history of these lineages, while lineage-specific expansions are concentrated at the terminal branches. *D. nepalensis* showed the largest number of gene family expansions (343) and a relatively higher number of contractions (218), suggestive of ecological specialization. Functional annotation of CAFE-significant families reveals that these changes are enriched in stress-response pathways—including chaperones, proteases, and chromatin-remodeling factors—and that cold-adapted species share convergent expansions in polyamine biosynthesis (ornithine decarboxylase [ODC]) and protein folding under stress (PDI, DnaJ, prolyl 4-hydroxylase), providing molecular evidence for cold-specialized ecological adaptation (John et al. 1993; Upadhyay et al. 2021). In contrast, the rest of the species, except *D. ananassae*, showed higher gene contraction events and lower gene family expansions. Overall, these patterns indicate that gene family evolution in this clade is characterized by a shared background of ancestral contraction, upon which each terminal lineage has superimposed distinct expansions. *D. nepalensis* shows the greatest net expansion among terminal branches (680 increases, 462 decreases), driven by functionally distinct gene family sets from those shared with other cold-adapted species, consistent with ongoing lineage-specific ecological specialization in its Himalayan environment rather than a single shared cold adaptation genomic program.

**Fig. 7.**
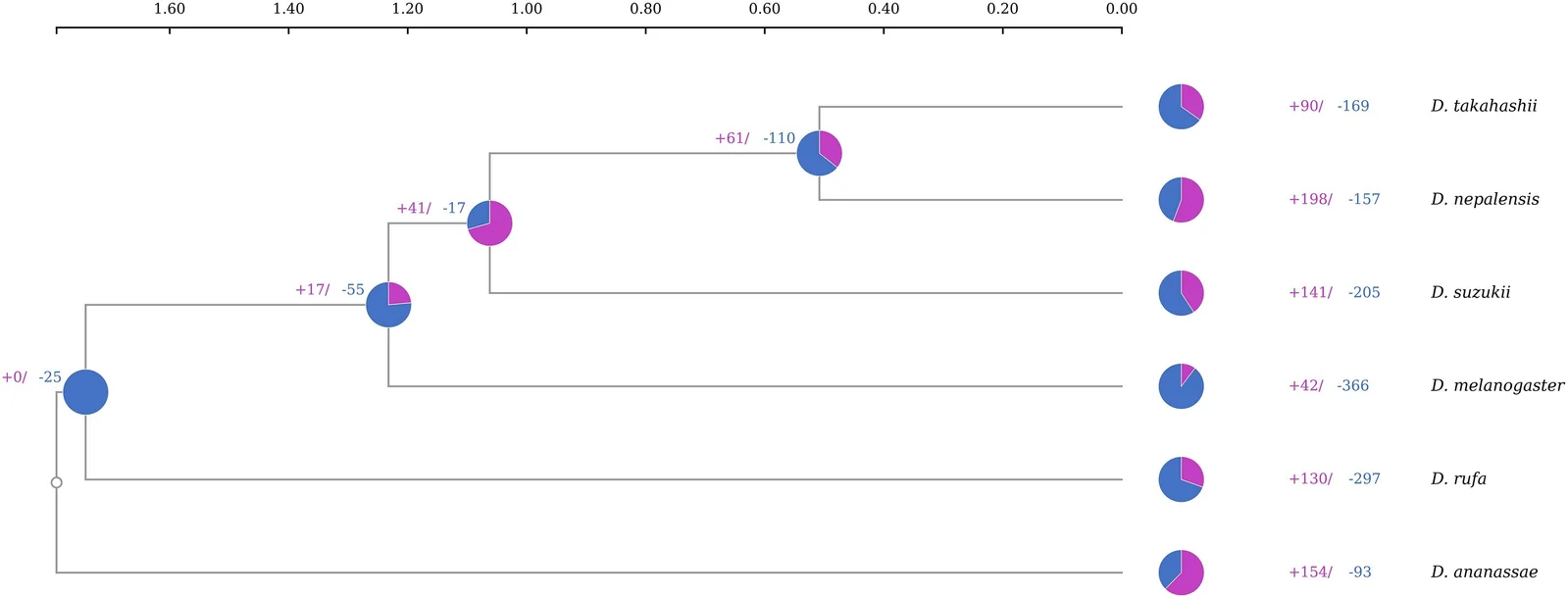
Evolutionary relationship of the 6 *Drosophila* species used for comparative genomics. Gene family expansion and contraction across 6 *Drosophila* species. The phylogenetic tree illustrates the inferred evolutionary relationships, with numbers in magenta indicating gene family expansions and numbers in blue indicating contractions along each lineage. Pie charts at each node represent the proportion of expanded (magenta) and contracted (blue) gene families.

#### Functional signatures of gene family expansion, contraction, and lineage specificity

Among the 83 gene families exclusive to *D. nepalensis*, 44% carried InterPro annotations. The most biologically notable included a caspase-family cluster (cluster2279; 7 genes; cysteine endopeptidase activity, GO:0004197), indicative of lineage-specific apoptotic regulation (Chen et al. 2026); clusters encoding High Mobility Group Box (HMGB) (Zhang and Wang 2010) and protamine-like chromatin-remodeling proteins (cluster11662; 5 genes) (Citterio et al. 2000); a MADF/Adf-1 transcription factor cluster (cluster12526; 4 genes) (Shukla et al. 2014); and a SNARE/syntaxin-domain cluster (cluster12420; 4 genes) associated with vesicle trafficking (Hong 2005).

Of the 198 gene families significantly expanded in *D. nepalensis* (CAFE5 *P* < 0.05) (Mendes et al. 2021), the most enriched domain class was the trypsin-type serine protease (S1 clan; Fig. 8), with 68 instances across expanded families—consistent with roles in immune defense and digestive adaptation (Supplementary Files 5 and 6). Additional notable expansions included a sodium:neurotransmitter symporter family (cluster169; Δ = + 9), a NADH:ubiquinone oxidoreductase Complex I subunit family (cluster244; Δ = + 6), and a TBP (TATA-binding protein) domain family (cluster112; Δ = + 13), suggesting lineage-specific divergence in neurochemical signaling, mitochondrial function, and transcriptional regulation, respectively.

**Fig. 8.**
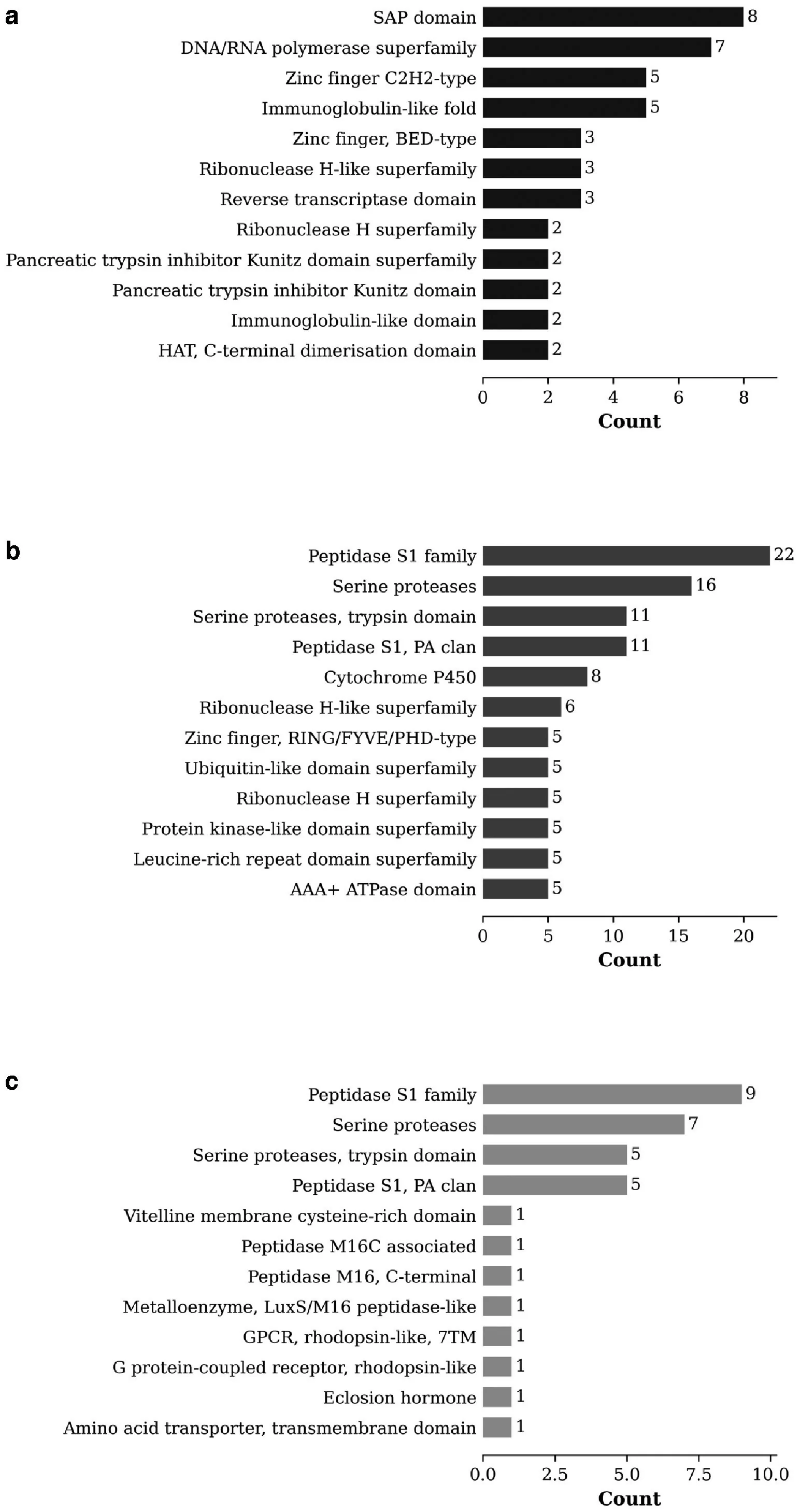
Functional characterization of lineage-specific and expanded gene families in *D. nepalensis*. a) Most frequent InterPro domains identified among orthogroups specific to *D. nepalensis*. b) Functional domains associated with significantly expanded gene families identified using CAFE analyses in *D. nepalensis*. c) Functional domains identified among expanded gene families associated with cold-associated species comparisons.

The 157 significantly contracted families in *D. nepalensis* were dominated by core histone gene families: Histone H4 (cluster2; Δ = −23), Histone H2B (cluster4; Δ = −19), Histone H2A (cluster5; Δ = −18), and Histone H3/CENP-A (cluster7; Δ = −18). These represent the largest magnitude changes in the entire dataset and were accompanied by expansions in HMGB/protamine-like families specific to *D. nepalensis*, pointing to a substantial reorganization of chromatin architecture in this lineage (Zhang and Wang 2010). Additional contracted families included Rho GTPase-activating proteins (cluster9; Δ = −13) and insect cuticle proteins (cluster59; Δ = −8).

Comparative analysis of significantly evolving families across cold-adapted species (*D. nepalensis*, *D. suzukii*, *D. rufa*) revealed no gene family expanded in all 3 species simultaneously, implying independent rather than shared ancestral expansion. Pairwise shared expansions were most numerous between *D. nepalensis* and *D. rufa* (49 families), including chaperone families (DnaJ, GroES, protein disulfide-isomerase/PDI), prolyl 4-hydroxylase (the conserved oxygen-sensing pathway orthologous to HIF-1α signaling and collagen stability pathway), and families carrying LOTUS/Tudor and Maelstrom domains (components of the piRNA TE silencing pathway). *D. nepalensis* and *D. suzukii* shared 20 expanded families, most notably an ODC family (cluster410), the rate-limiting enzyme in polyamine biosynthesis (Kapfhamer et al. 2020)—a pathway with well-established roles in cryoprotection in cold-tolerant insects (Walters et al. 2009; Purać et al. 2015). All 3 cold-adapted species shared 25 contracted families, the majority representing minor losses, though including a zinc finger CCHC-type family and a serine protease family.

## Discussion

This is the first study to report a high-quality reference genome for *D. nepalensis*, which is of great importance in understanding the cold-adapted drosophilids that inhabit montane ecosystems and are endemic. The substantial coverage obtained from both sequencing platforms, Illumina and Nanopore, ensured robust base-level accuracy of the assembled genome, which allows for downstream annotation and analysis. This hybrid assembly and comprehensive annotation revealed the expansion of stress-response domains, such as zinc-finger proteins (Li et al. 2024; Miao et al. 2025), and a significant representation in oxidative stress and lipid metabolism pathways—traits that are concordant with those of cold-adapted insects (Storey and Storey 2012; Purać et al. 2015).

The thermal preference assays for the 3 species, *D. melanogaster*, *D. ananassae*, and *D. nepalensis*, revealed a pronounced bias in *D. nepalensis* toward the colder zones over the hot and medium zones. There is relatively higher occupancy of the hot and medium zones by *D. melanogaster* and *D. ananassae*, both of which are cosmopolitan species (Markow and O'Grady 2007; Singh 2025). This is consistent with their broader climatic distribution. In contrast, the distribution of *D. nepalensis* is restricted to high-altitude and colder environments. These behavioral differences lay the groundwork for interpreting the genomic signatures of *D. nepalensis* in relation to other cold-adapted and cosmopolitan taxa within a comparative genomics framework.

The annotation of the *D. nepalensis* genome revealed a rich landscape of repetitive elements, indicating that 27.01% of the assembly is comprised of repeats, which is consistent with the assembled high-quality genomes of other Drosophilids (Flynn et al. 2023; Wang et al. 2025). LINE and LTR retrotransposons predominate, while a marked contraction is noted for certain DNA transposon families (PiggyBac, En-Spm, MULE-MuDR), hinting at a lineage-specific contraction event often linked to adaptation in stressful environments (Tahami et al. 2024). The significant proportion of unclassified repeats (12.44%) further points to an underexplored TE diversity in Himalayan drosophilids.

The annotation of 10,560 protein-coding genes, along with 233 tRNA and 43 rRNA genes, underscores the structural and functional completeness of the genome. InterPro scan revealed the dominance of regulatory, enzymatic, and structural domains, typical of any insect genome. Notably, the highest abundance of zinc finger C2H2-type domains (4,939) suggests a considerable repertoire of transcription factors and DNA-binding proteins—known for their functions in response to abiotic stressors and development (Li et al. 2024; Miao et al. 2025). The prevalence of WD40 repeats, P-loop nucleoside triphosphate hydrolases, and serine proteases further reflects the adaptive complexity of the genome of *D. nepalensis*, which is necessary for adaptation to stressful, montane habitats (Sharma and Pandey 2015; He et al. 2018; Gao et al. 2019).

GO term enrichment analyses revealed that protein binding, membrane association, proteolysis, and ATP binding are dominant molecular functions and biological processes in the *D. nepalensis* proteome. Cold adaptation was reflected by the representation of chitin-binding and short-chain dehydrogenases/reductases (SDR) domains (Koenekoop et al. 2022), supporting mechanisms for cuticle integrity (Tao et al. 2023) and redox/lipid metabolism (Wu et al. 2023). Cold adaptation–related GO terms were also prominent, most notably those involved in oxidoreductase activity, membrane organization, fatty acid metabolism, and response to stress, consistent with other insect genomes adapted to colder habitats (Parker et al. 2021; Vatanparast and Park 2021; Enriquez and Teets 2025).

The comparative orthology analysis revealed evolutionary conservation within the *Drosophila* genus, with 9,173 orthologous clusters forming the core genomes shared by all 6 species (*D. melanogaster*, *D. ananassae*, *D. suzukii*, *D. takahashii*, *D. nepalensis*, and *D. rufa*). This conservation elucidates the stability of the essential developmental, metabolic, and regulatory pathways, reflecting patterns observed in any comparative genome studies as well (Paiva et al. 2024; Wen et al. 2025). Beyond this core, 1,716 orthogroups are shared among subsets of species, pointing to lineage-specific gene expansion or contraction events during divergence. While *D. nepalensis* and *D. rufa* share a subset of CAFE-significant expanded gene families (*n* = 49 pairwise, enriched for chaperone and protein folding functions) and contracted families (*n* = 61 pairwise, including core histone gene families), no gene family was simultaneously expanded in all 3 cold-adapted species, suggesting that convergent cold adaptation at the genomic level has occurred through independent expansions of partially overlapping functional pathways rather than a shared ancestral cold adaptation gene set.

Relatively higher singleton counts in *D. nepalensis* (50) and *D. rufa* (46) suggest a greater number of lineage-specific genes in these species; however, this pattern may reflect some combination of gene innovation, divergence, and demographic processes such as drift in addition to adaptation. These unique orthogroups and singletons might indicate endemicity and ongoing adaptation to high-altitude environments. Similar patterns have been documented in *Drosophila montana*, where population genomic diversity was associated with cold adaptation and habitat seasonality (Parker et al. 2018; Wiberg et al. 2021). Gene families related to ion transport and membrane stability are repeatedly highlighted as important regulators of overwintering and reproductive diapause in cold-tolerant species (Enriquez and Colinet 2019; Lee 2021; Bao et al. 2023), which are also well-represented in the *D. nepalensis* genome.

The gene family evolution analysis indicates both expansion and contraction events in *D. nepalensis*, contrasting with a majority of contractions in cosmopolitan species. The substantial expansion and contraction of gene families in *D. nepalensis* suggest that it is getting specialized to only a certain kind of environment, mainly cold, which is further suggestive of the subsequent restriction of its range, rendering it endemic. It may have lost the potential to spread or adapt to other habitats, allowing it to move only upward to colder habitats as its native elevations become hotter due to climate change (Parkash et al. 2013). Similar gene family expansion events have been shown to be responsible for cold adaptation (Kim et al. 2022). Also, contraction events leading to host specialization have been reported in other taxa (Baroncelli et al. 2016; Rogers et al. 2022). In another study, gene family contraction has led to adaptations in metabolism, osmoregulation, and developmental regulation in a fish genome, reflecting genomic responses to ecological specialization (Zhang et al. 2022). Moreover, gene family expansions have led to genomic innovations leading to ecological specialization across taxa (Kim et al. 2022). Thus, the advent of both expansion and contraction events might have been responsible for the ecological specialization of *D. nepalensis*, owing to faster protein evolution rates, suggestive of rapid functional divergence (Prachumwat and Li, 2008). Specifically, the dramatic contraction of histone gene families (H2A, H2B, H3, H4; Δ = −18 to −23 genes) alongside the *D. nepalensis*–specific expansion of HMGB/protamine-like chromatin remodelers suggests that chromatin architecture has been substantially restructured in this lineage—a form of genomic specialization that could underpin altered transcriptional programs at low temperatures. The convergent expansion of polyamine biosynthesis genes (ODC) between *D. nepalensis* and *D. suzukii*, and of chaperone and prolyl 4-hydroxylase families between *D. nepalensis* and *D. rufa*, provides the first functional evidence that cold specialization in Himalayan drosophilids involves protein homeostasis and cryoprotective metabolite production as convergently evolved molecular strategies.

The *D. nepalensis* genome assembly achieved 98% BUSCO completeness (Fig. 9), matching established *Drosophila* models and exhibiting a high ANI with other *Drosophila* species, confirming both robust gene content and overall genome conservation, while also capturing lineage-specific divergence. The integration of cold-adapted and cosmopolitan *Drosophila* genomes provides new insights into the evolutionary basis of thermal adaptation. The combination of a large conserved core genome with lineage-specific differences in gene family size reflects a pattern of genomic stability in essential pathways alongside copy-number variation in gene families associated with proteolysis, chromatin regulation, and molecular chaperone function in *D. nepalensis.*

**Fig. 9.**
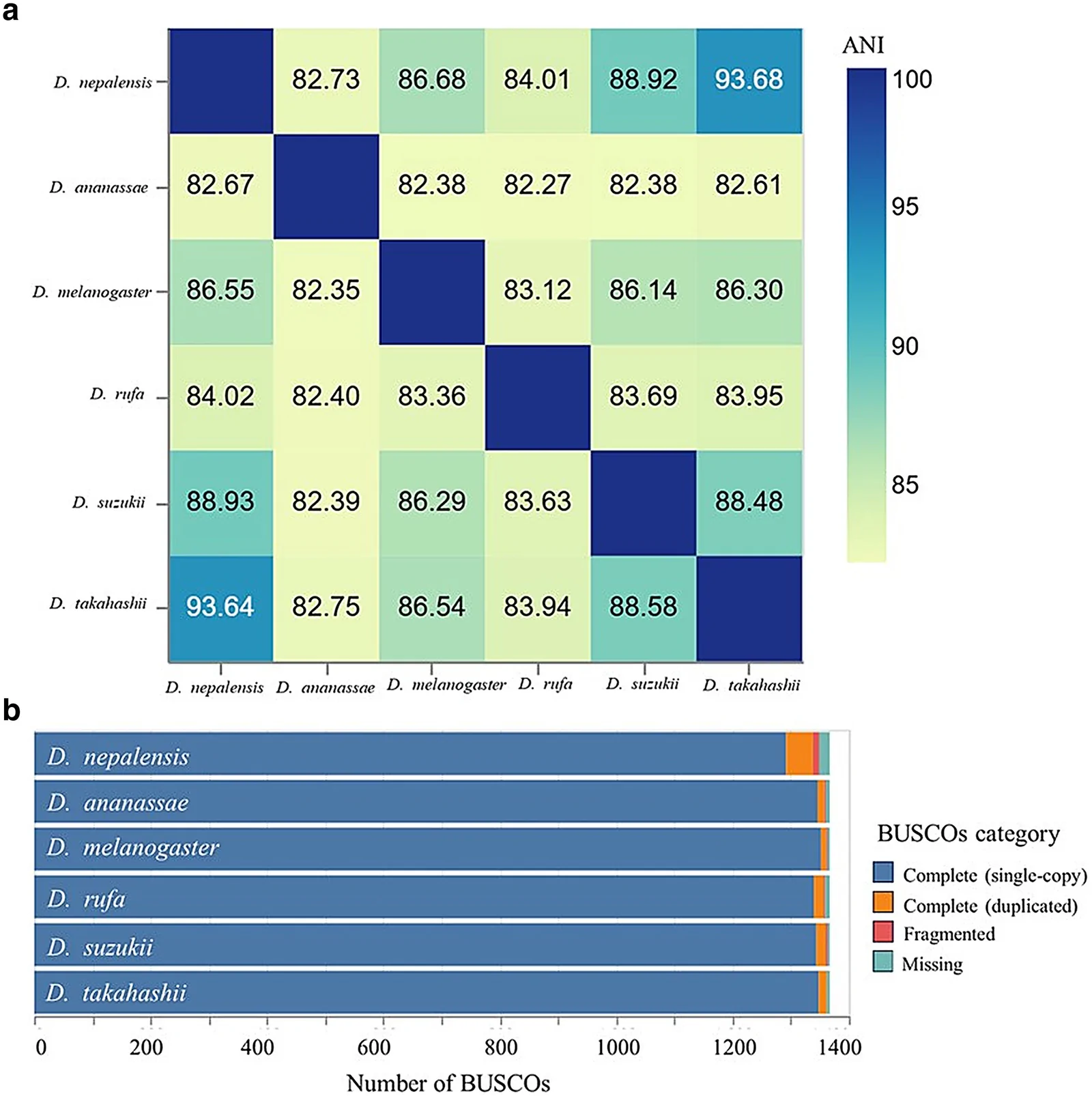
Comparative genomics of 6 *Drosophila* species, *D. ananassae*, *D. melanogaster*, *D. nepalensis*, *D. rufa*, *D. suzukii*, and *D. takahashii*, and based on their assembled genome. a) ANI heatmap of the assembled genomes from 6 species used in comparative genomics. Darker colors indicate higher ANI, while lighter colors show lower identity. b) BUSCO comparison among the 6 species. The BUSCO comparison among the 6 species was done by MOSGA2 using the assembled genomes of the 6 species with the insecta_odb10 database. *D. nepalensis* has the highest number of complete (duplicated) and fragmented categories due to the first assembly, which has lower coverage.

Future directions include integrating transcriptomic, epigenomic, and population genomic datasets to elucidate the functional roles of novel and expanded gene families. Linking genomic variation with phenotypic plasticity and thermal adaptation profiles will provide a more mechanistic understanding of cold adaptation. Furthermore, comparative expression analyses across temperature gradients could identify regulatory shifts driving ecological specialization. Such efforts will be essential for predicting the resilience of montane drosophilids, such as *D. nepalensis*, to ongoing climate change.

## Supplementary Material

jkag190_Supplementary_Data

## Data Availability

All raw sequencing reads (FASTQ format) from both Illumina and Nanopore platforms, along with the assembled genome, have been deposited in the NCBI Sequence Read Archive (SRA) under BioProject accession number PRJNA1415802. Data are also available at GSA FigShare: https://doi.org/10.25387/g3.31316818. Supplemental material available at G3 online.
